# A P300-Based Brain-Computer Interface for Improving Attention

**DOI:** 10.3389/fnhum.2018.00524

**Published:** 2019-01-04

**Authors:** Mahnaz Arvaneh, Ian H. Robertson, Tomas E. Ward

**Affiliations:** ^1^Department of Automatic Control and Systems Engineering, University of Sheffield, Sheffield, United Kingdom; ^2^Global Brain Health Institute, Institute of Neuroscience, Trinity College Dublin, Dublin, Ireland; ^3^Insight Centre for Data Analytics, School of Computing, Dublin City University, Dublin, Ireland

**Keywords:** brain-computer interface, neurofeedback, P300, attention, electroencephalography

## Abstract

A Brain-computer Interface (BCI) can be used as a neurofeedback training tool to improve cognitive performance. BCIs aim to improve the effectiveness and efficiency of the conventional neurofeedback methods by focusing on the self-regulation of individualized neuromarkers rather than generic ones in a graphically appealing training environment. In this work, for the first time, we have modified a widely used P300-based speller BCI and used it as an engaging neurofeedack training game to enhance P300. According to the user's performance the game becomes more difficult in an adaptive manner, requiring the generation of a larger and stronger P300 (i.e., in terms of total energy) in response to target stimuli. Since the P300 is generated naturally without conscious effort in response to a target trial, unlike many rhythm-based neurofeedback tools, the ability to control the proposed P300-based neurofeedback training is obtained after a short calibration without undergoing tedious trial and error sessions. The performance of the proposed neurofeedback training was evaluated over a short time scale (approximately 30 min training) using 28 young adult participants who were randomly assigned to either the experimental group or the control group. In summary, our results show that the proposed P300-based BCI neurofeedback training yielded a significant enhancement in the ERP components of the target trials (i.e., 150–550 ms after the onset of stimuli which includes P300) as well as attenuation in the corresponding ERP components of the non-target trials. In addition, more centro-parietal alpha suppression was observed in the experimental group during the neurofeedback training as well as a post-training spatial attention task. Interestingly, a significant improvement in the response time of a spatial attention task performed immediately after the neurofeedback training was observed in the experimental group. This paper, as a proof-of-concept study, suggests that the proposed neurofeedback training tool is a promising tool for improving attention particularly for those who are at risk of attention deficiency.

## 1. Introduction

Brain-computer Interface (BCI) provides a direct communication pathway between the human's brain and an external device (Wolpaw et al., 2002; Birbaumer, 2007). Using appropriate sensors and computational algorithms, BCI analyses brain signals, extracts relevant brain patterns and translates them to control signals. The development of BCI was initially focused on severely paralyzed people, helping them gain some sort of independence by controlling assistive devices using their thoughts (Wolpaw et al., 2002; Birbaumer, 2006). Recently, a number of new applications of BCI targeting other user groups have attracted attention. For example, BCI has been considered as a rehabilitation tool encouraging neuroplasticity in stroke survivors (Ang et al., 2011, 2015). Moreover, BCI has been suggested as a potential neurofeedback training tool for improving cognitive performance (Lim et al., 2012; van Erp et al., 2012; Tih-Shih et al., 2013; Yang et al., 2018).

Different aspects of neural activities could be very well associated with different cognitive, behavioral or emotional states such as attention, memory, mood, etc. As a neurofeedback training tool, BCI needs to measure changes in relevant neural markers that have implication on the intended emotional states or cognitive performance, and subsequently provide feedback about them to the user. Thus, by providing real-time feedback, a BCI can encourage the user to alter those neural markers toward optimal patterns associated with a superior performance.

Previous studies showed that it is possible to influence certain frequencies of the electrical activity of the brain measured via scalp electrodes (a method termed electroencephalography or EEG) such as theta, alpha, alpha/theta ratio, beta, gamma and sensorimotor rhythms for improving attention, memory, complex motor skills, procedural learning, perceptual binding, cognitive processing speed, intelligence, and mood (Vernon et al., 2003; Angelakis et al., 2007; Wang and Hsieh, 2013; Gruzelier, 2014). While some promising outcomes were reported, conventional neurofeedback training is typically repetitive, monotonous and boring. Some participants undergo several trial and error attempts during many sessions before they have some success in self-regulating the desired brain patterns. BCI-based neurofeedback training aims to mitigate these limitations and make the training more effective and efficient by (1) integrating neurofeedback in an engaging game environment and (2) using individualized neuromarkers rather than generic neuromarkers extracted by machine learning techniques (Thomas et al., 2013; Tih-Shih et al., 2013; Ordikhani-Seyedlar et al., 2016; Ordikhani-Seyedlar and Lebedev, 2018).

In this study, we have modified the widely used P300-based BCI speller paradigm in order to develop a neurofeedback training tool capable of enhancing the P300. Generally, P300 is a positive deflection in the EEG signal that appears approximately 300 ms after the presentation of an attended stimulus (Sutton et al., 1965). Interestingly, P300 is known as a valuable tool for assessing cognitive function. P300 components (e.g., amplitude, latency, energy) reflect information processing associated with attention, speed of stimulus processing, error awareness and memory performance (Gonsalvez and Polich, 2002; Pourtois et al., 2006; Polich, 2007). According to several research studies, shorter P300 latency and larger P300 amplitude are associated with superior with superior performance in the above-mentioned cognitive functions (e.g., Polich, 2007). This association is also supported by results indicating that both age and dementia lead to reductions in the energy and amplitude of P300 as well as an increase in the latency of P300 (Ashford et al., 2011; van Dinteren et al., 2014).

The P300-based BCI speller was proposed and implemented by Farwell and Donchin Farwell and Donchin (1988), and since then has been widely used in the BCI community as a communication tool. In a P300-based BCI, a series of repeating stimuli (e.g., letters) are presented on a screen to the user. For item selection the user is required to attend the desired associated stimulus and ignore the rest. At the end of each sequence, the BCI identifies which stimulus was attended as P300 is expected to be generated for the target stimulus if well attended. Unlike motor imagery-based BCIs, the P300-based BCI speller can be controlled with high accuracy using a very short training time (Guger et al., 2009). Indeed, previous studies showed that almost all healthy people and many patients are able to operate this type of BCI (Sellers and Donchin, 2006; Guger et al., 2009).

This study proposes a new version of the P300-based speller BCI as an interactive neurofeedback training game, such that according to the user's performance the game becomes more difficult requiring the generation of a larger amplitude P300 for target stimuli. For this purpose, the sequence of presented stimuli gets shorter by reducing the number of repetitions, leading BCI to identify P300 waves by averaging across a smaller number of EEG trials. Thus, to have a correct detection by BCI, the user needs to increase the stability of his/her P300 responses, since a noisy less attended target trial has a more negative influence on the BCI detection output. The reduction in the number of trials per sequence is done according to the user's performance to avoid frustration arising from task difficulty.

According to previous research, human errors caused by lapses of attention and mind wandering are known to degrade the performance of P300-based BCIs (Fazel-Rezai, 2007; Lakey et al., 2011). In addition, modulation of P300 components in a BCI system depends on psychological factors such as motivation, mental fatigue, sustained attention, frustration and anxiety caused by performance errors (Kleih et al., 2010; Kleih and Kübler, 2015). The proposed P300-based BCI aims to enhance the generated P300 since providing more frequent feedback possibly helps avoid lapses in attention and mind wandering and increases the user's motivation and task engagement as suggested in Finke et al. (2009), Ganin et al. (2013), and Arvaneh et al. (2015).

To the best of our knowledge, the present paper is the first study that investigates the self-regulation of P300 through neurofeedback training. We hypothesize that the proposed tool has the potential to be used for improving attention through enhancing P300. To test this hypothesis, two groups of participants (i.e., control and neurofeedback) are employed. We first quantitatively investigate whether or not the proposed P300-based BCI changes the components of P300 toward patterns associated with a superior cognitive performance (Polich, 2007). Thereafter, we compare cognitive performance of the participants before and after playing the proposed P300-based neurofeedback game using a visual perception task referred to as the random dot motion (RDM) task (Kelly and O 'connell, 2013). The RDM task has been extensively used in decision making studies, and its performance can be an indicator of the user's spatial attention (Sapir et al., 2005).

## 2. Methods

### 2.1. Participants

In total, thirty healthy young adults (12 male, 18 female) aged 20–39 years participated in the study. The participants had normal or corrected-to-normal vision, and had no history of cardiac disease and neurological illness (such as seizure, brain injury, stroke). The participants gave written informed consent for the study which had been reviewed and approved by the ethical review board of the School of Psychology, Trinity College Dublin, in accordance with the declaration of Helsinki.

Participants were randomly assigned to an experimental group and a control group. Two females, one from each group, were excluded due to a very poor performance in the Continuous RDM task (i.e., ≤ 35% correctly detected targets) and a very large average total power of the non-target EEG trials in the P300-based speller BCI task (i.e., the difference to the grand average was greater than three standard deviations), respectively. As a result, the final sample for the experimental group consisted of 14 subjects (7 female) with a mean age of 27.85 (SD ± 4.91); and the final sample for the control group consisted of 14 subjects (9 female) with a mean age of 25.57 (SD ± 3.50). 22 out of 28 participants did not have any experience of using BCI. The remaining 6 participants (4 in the experimental group and 2 in the control group) had previously attended a P300-based BCI speller study as participants. There were no significant differences between the two groups according to the demographic variables [age: *t*_(26)_ = −1.42, *p* = 0.17; gender: $\chi_{\left(1\right)}^{2}=0.58,p=0.44$; and previous BCI experience: $\chi_{\left(1\right)}^{2}=0.848,p=0.35$]. Furthermore, both groups were very successful in using the P300-based BCI task for spelling, as the control and experimental groups achieved average classification accuracies of 95.238±12.10 and 97.62±8.90, respectively in the evaluation phase.

### 2.2. Procedure

**Figure 1:**
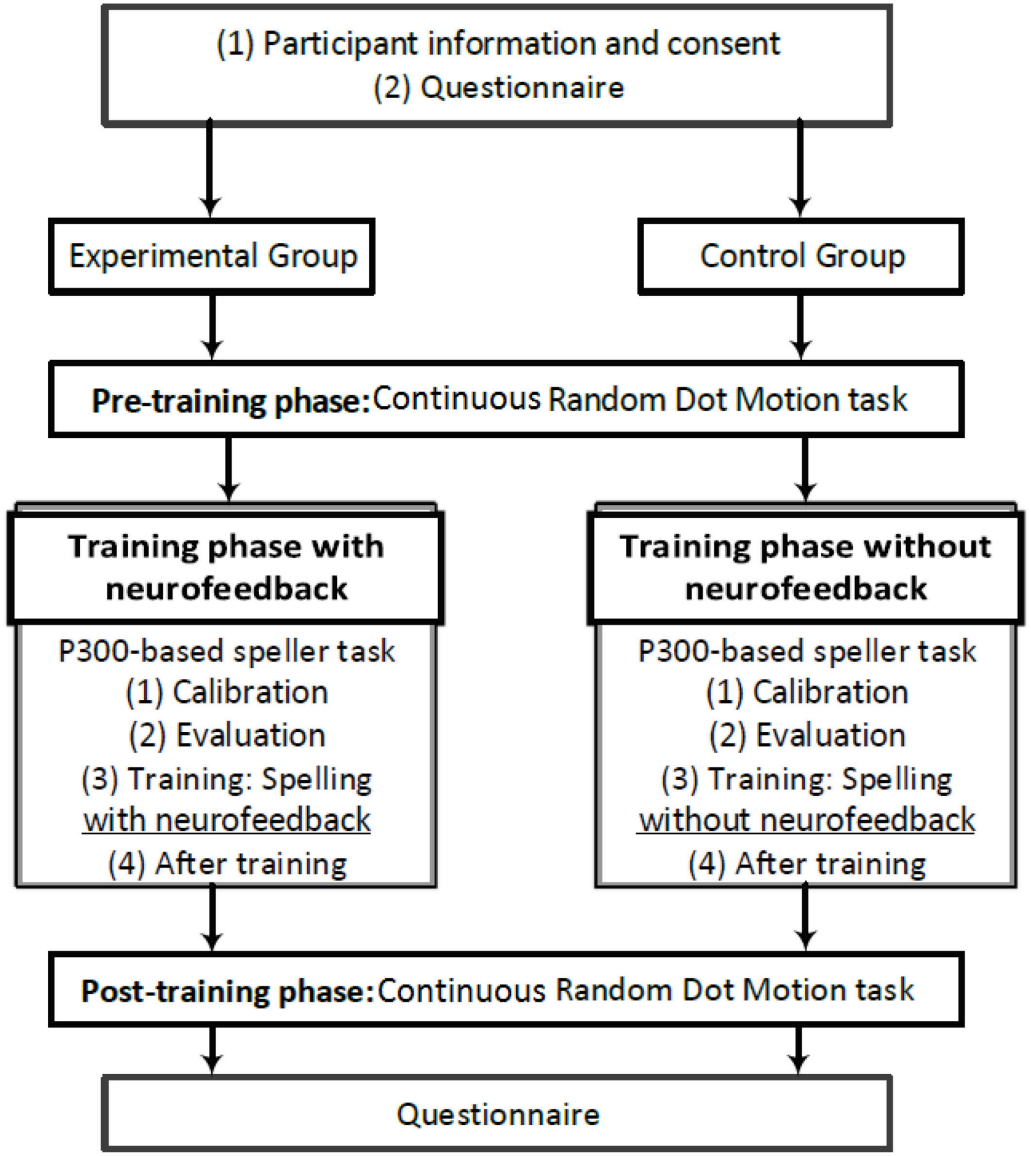
A schematic illustration of the procedure in the present study.

The procedure of the present study is illustrated in Figure 1. Participants visited the laboratory for one session, which lasted less than 1 h and 45 min including the set up time. The experiment took place in a dark, sound-attenuated, closed room.

At the beginning and at the end of the session, the participants filled out a simple questionnaire asking about their tiredness and boredom. To investigate the effects of the proposed neurofeedback training on cognitive performance, prior to and after the training phase, some aspects of the participants' cognitive abilities were assessed using a continues version of the classic random dot motion (RDM) task, while EEG data were recorded.

During the training phase, the participants performed a P300-based speller task. The details of this task is explained in section 2.3.3. The initial EEG data collected at the beginning of the P300-based Speller task were used for calibrating a subject-specific model which distinguishes between the target and the non-target EEG trials. After evaluating the model on some new EEG trials and being ensured of its satisfactory performance, the participants were asked to perform the P300-based speller task again to spell some new letters. In this stage, those who were in the experimental group received regular feedback based on their EEG components (i.e., P300), whereas the control group did not receive any feedback.

### 2.3. Tasks and Stimuli

#### 2.3.1. Questionnaire

The questionnaire consists of four items to be answered, namely (1) How tiered you are now? (2) How alert do you feel? (3) How bored do you feel? (4) Do your eyes feel tiered? The participants were required to report their level of fatigue, alertness, boredom, and tiredness of their eyes on a 10-point likert scale ranging from low to high.

#### 2.3.2. Pre- and Post-training Continuous RDM Task

**Figure 2:**
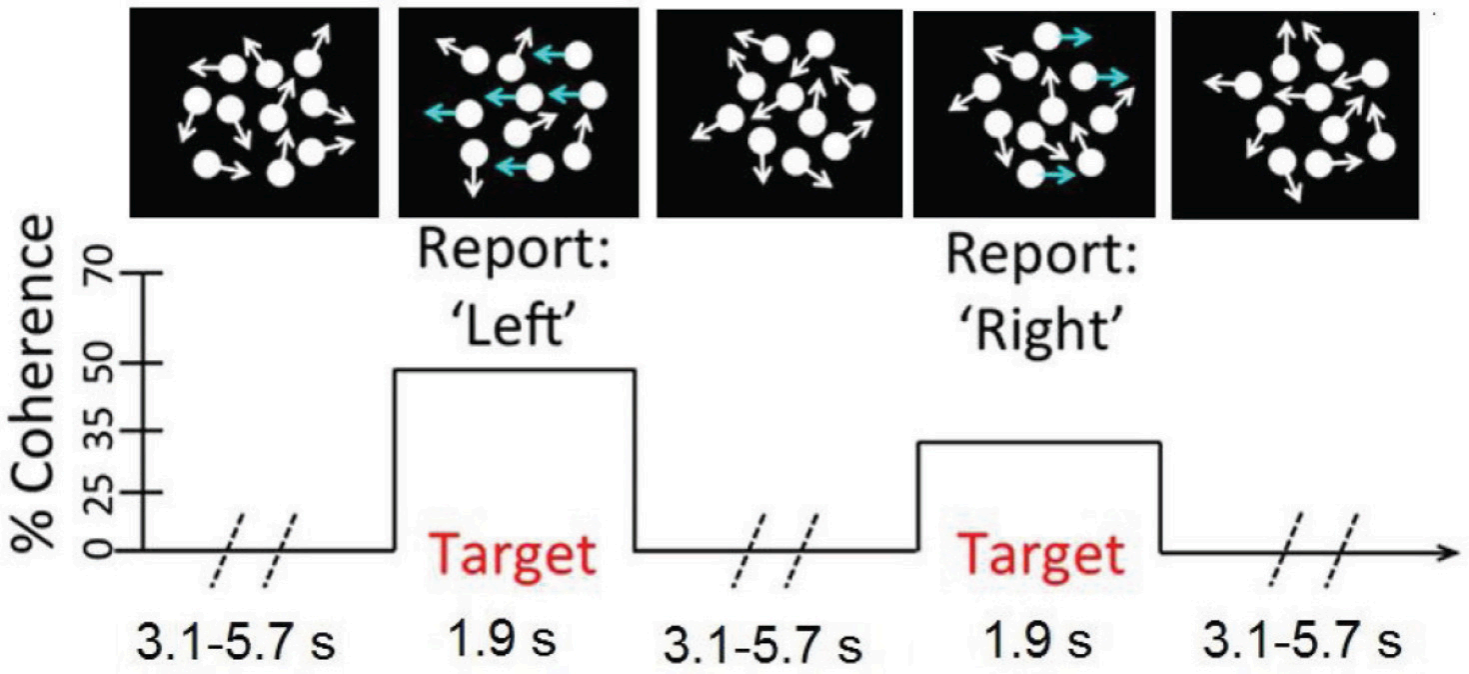
A schematic of the continuous RDM task. Participants monitored a number of centrally presented dots for step transitions from random to coherent motions.

We used a continuous version of the classic RDM task which is very similar to the task described in Kelly and O 'connell (2013). In this task, the participants monitored a patch of incoherently moving dots for step transitions from incoherent to coherent motions in either the leftward or the rightward direction. As can be seen in Figure 2, in the incoherent motions, the dots are moving in all directions with no overall motion vector, whereas in the coherent motions a fraction of the dots are moving coherently in the same direction. The larger the level of motion coherence, the greater is the proportion of the dots moving unambiguously in one direction. The participants were instructed to respond to the leftward motion with a left-hand button press and to the rightward motion with a right-hand button press as soon as they were sure of the motion direction. The motion direction and the coherence level varied independently and randomly on a target-by-target basis.

In this study the coherent motion lasted for a period of 1.9 s, and the coherence level took one of the two values: 19, 25%. The inter-target interval, during which the incoherent motion was continuously displayed, lasted 3.1, 4.2, or 5.7 s, chosen randomly on a trial-by-trial basis. A total of 40 targets were presented in each pre-training and post-training phase. The visual stimuli were presented against a dark gray background on a cathode ray tube (CRT) monitor operating at a refresh rate of 85 Hz and resolution of 1, 024 × 768. There were a total number of 118 white dots (each 6 × 6 pixels) within the circular aperture, which moved on every 47 ms.

Participants completed three short practice sessions before starting the pre-training phase. Each practice session contained 6 target trials. In the first practice, all the targets were presented at the coherence levels of either 80 or 60% to ensure that each participant understood the nature of the task. In the second practice session, the coherence levels were 40 and 30%, and in the third practice session, the coherence levels were 25 and 20%. During the practice sessions, verbal feedback was provided on hits, misses, and false alarms, helping the participants to better understand the task.

#### 2.3.3. P300-Based Speller Task

A traditional brain-computer interface (BCI) P300-based speller task was used as an interactive interface to present stimuli and provide neurofeedback. The P300-based speller task was proposed and implemented by Farwell and Donchin in 1988 (Farwell and Donchin, 1988), and since then it has been widely used in BCI community as an assistive device for communication. In this study, we modified this interface, and used it for a new purpose; i.e., neurofeedback training. Specifically, the task was modified to become more difficult based on the participant's performance, with the aim of encouraging the participant to generate larger and stronger P300 waves on the target stimuli.

**Figure 3:**
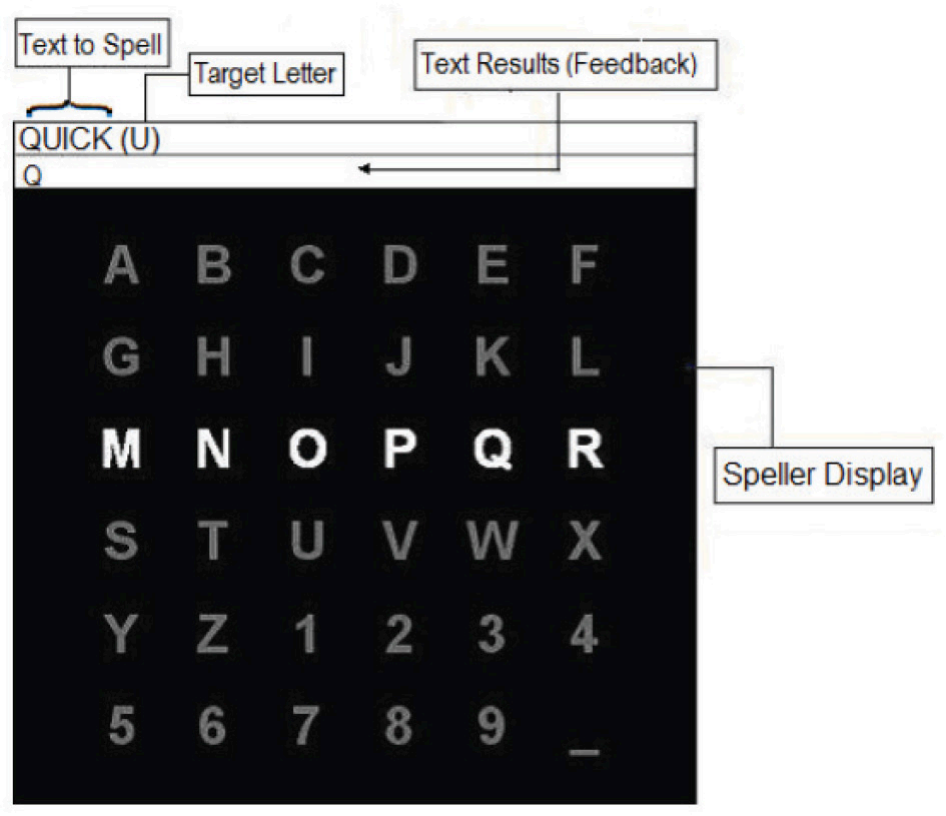
The interface of the P300-based speller task.

Figure 3 presents the interface of the applied P300-based speller. A 6 × 6 matrix, containing the letters of the alphabet and other symbols, was displayed on a computer screen, while EEG signals were recorded. The text-to-spell was displayed above the matrix. Directly next to the text-to-spell, the target letter-to-spell was being presented in the parenthesis. The rows and the columns of the matrix were flashed/intensified in a random order. Each flash lasted 55 ms followed by an inter stimulus interval of 117 ms. The participants were instructed to concentrate on the target letter and silently count how often it is flashed. The flashes of the row and the column containing the target letter (i.e., target stimulus) resulted in EEG signals which should contain P300 components, while the flashes of the other rows and columns (i.e., non-target stimuli) corresponded to non-target EEG signals. Thus, the target letter could be inferred by a simple classification algorithm that searches for the row and the column which evoked the largest P300 features.

In this study, the P300-based speller task was initialized with 12 flashes per row/column. Subsequently, the EEG response to each row/column was obtained by averaging over the 12 corresponding EEG trials. The averaging increases the signal to noise ratio as the P300 wave may co-exists with ongoing EEG activity unrelated to the task obscuring the required signal. After one sequence, consisting 144 (i.e., 12 × 12) target and nontarget flashes in total, the matrix stopped flashing for 6 s. During this interval, the next target letter-to-spell was displayed within the parentheses, and the participant was given time to locate it in the matrix. In addition, in the neurofeedback phase, the letter identified by the classifier also appeared in the text results (feedback) box. Indeed, the feedback provided indicated whether or not the participant's generated EEG signals to the target stimuli were correctly distinguished from non-target stimuli. These steps were repeated until the entire text was successfully spelled out.

#### 2.3.4. Protocol of the Training Phase

The proposed training phase involved four stages:

##### 2.3.4.1. Calibration

In this stage, the participants completed two runs, in which the words “the" and “quick" were respectively spelled without providing feedback. The sequence of attending each letter consisted of 12 flashes per row/column. The EEG data collected from this stage were used to calibrate a subject-specific model identifying attended letters.

After completing the two runs, the EEG responses to each row/column was obtained by averaging over the 12 corresponding EEG trials. Thereafter, 400 ms segments starting 150 ms after the onset of the stimuli were extracted from the EEG responses. We focused on this time interval in order to take into account all the possible effects of the P300 in our model. The segments were then moving average filtered and decimated to 20 Hz. The resulting data arrays obtained from the applied electrodes then concatenated and created the feature vector. The dimension of the feature vector was *N*_*e*_×*N*_*t*_, where *N*_*e*_ denotes the number of electrodes and *N*_*t*_ denotes the number of temporal samples in an EEG response. Due to considering the duration of 400 ms and the down-sampling to 20 Hz, *N*_*t*_ always equaled to 8. The extracted features were then processed using a linear discriminant analysis (LDA) classifier to derive the EEG weighting parameters. The LDA weights each feature according to its importance, such that the distance between the means of the two classes (i.e., target and non-target trials) is maximized and the inter-class variance is minimized (Fukunaga, 2013). The great advantage of this technique is its low computational time and its simplicity. Moreover, its reliable performance has been presented in several BCI P300-based speller studies (Lotte et al., 2007).

##### 2.3.4.2. Evaluation

In this stage, the participants spelled the word “dog" without receiving feedback. The EEG data collected from this stage were used to evaluate the model calibrated in the calibration stage. For this purpose, the EEG features were extracted as explained previously. Thereafter, the trained LDA classifier was applied on the extracted features to identify the target letters. If at least two of the three letters of “dog" were identified correctly, the participant was ready to move to the training stage. Otherwise, the model was re-calibrated by adding the EEG data collected from spelling “dog". Thereafter, a new word, such as “fox", was spelled for evaluating the new model. If still the model did not reach the desired performance (i.e., correctly identifying minimum 2 out of 3 target letters), the participant was removed from the study. It is good to note that in this study all the participants were able to achieve a calibration model with satisfactory performance.

##### 2.3.4.3. Training

In this stage, the participants spelled the word “beautiful" in either four or five runs (see below for more details). For the experimental group, the calibrated model provided feedback at the end of each sequence of flashes by identifying the target letter based on the collected EEG signals. Indeed, the feedback encourages the participant to achieve a high spelling performance by maintaining/increasing the differences between the target and the non-target EEG trials. Since the calibrated model identifies the target letters by comparing the EEG responses around 300 ms after the onset of the stimuli, the feedback indirectly encourages maintaining/increasing the strength of the evoked P300 waves on the target trials while minimizing the Event-related potential (ERP) components on the nontarget trials. Importantly, based on the participants' performance, the task adaptively got more difficult by reducing the number of flashes per row/column in each sequence. Thus, to achieve a good performance, the participant required to generate stronger P300 in every target flash (stimulus), since the decision made by the calibration model was getting more dependent on the strength of each individual P300 response.

The number of flashes in the first run, denoted as *N*_1_, was set to 10. The number of flashes in the following runs (*N*_*i*_, *i* ∈ ℕ, *i*≥2) were defined as follows: Assume *N*_*i*−1_ denotes the number of flashes applied per row/column in the previous run. The accuracies of identifying the previously spelled word were calculated using different number of flashes per row/column ranging from 1 to *N*_*i*−1_. Subsequently, the minimum number of flashes that yielded an accuracy higher than 66% in the previous run was found (denoted as *N*_(*i*−1)66_). Finally, the number of flashes in the new run, *N*_*i*_, was set to the average of *N*_*i*−1_ and *N*_(*i*−1)66_. If the maximum accuracy obtained in the previously spelled word was less than 66%, the number of flashes in the new run was increased by one (i.e., *N*_*i*_ = *N*_*i*−1_+1), in order not to make the task too frustrating for the participant. The training stage terminated after four runs if $\sum_{i=1}^{4}N_{i}\geq 40$, otherwise the training stage terminated after 5 runs. This rule allowed us to have nearly similar training times for both groups.

The participants in the control group underwent entirely similar process, but without receiving any feedback. They were not informed about the output of the LDA classifier neither on the screen nor orally. They were not also informed that the changes in the number of flashes in each sequence is associated with their performance.

##### 2.3.4.4. After training

In this stage, both the experimental and control groups spelled the word “dance" based on 12 flashes per row/column without receiving feedback . The aim of this stage was to investigate the differences in the generated brain patterns between the two groups after training.

### 2.4. EEG Data Acquisition

EEG was acquired through the high impedance ActiveTwo Biosemi system from 8 electrodes located at positions Fz, C3, Cz, C4, P3, Pz, P4, and Oz following the international 10–20 standard system (Sharbrough et al., 1991). The impedances were kept below 5kΩ and the sampling rate was 512 Hz. The applied P300-based speller task and the continues RDM task were designed using the BCI2000 software platform (Schalk et al., 2004) and MATLAB, respectively. The EEG acquisition took place in a sound-attenuated room.

The online analysis of the EEG data were done as explained in section 2.3.4 using BCI2000. Further offline analysis was also conducted using MATLAB to investigate the impact of the proposed neurofeedback training protocol. In the offline analysis, the EEG data were re-referenced to Fz, and filtered with a zero-phase-shift low-pass 35 Hz Butterworth filter and a zero-phase-shift high-pass 0.5 Hz Butterworth filter. The EEG data were segmented and baseline corrected relative to the interval –150 to 0 ms before the onset of the stimuli. Segments with amplitudes exceeding +75μ*V*, or voltage steps of more than 150 μ*V* within a window of 200 ms were rejected from further analysis.

### 2.5. Data Analysis

#### 2.5.1. Pre- and the Post-experiment Questionnaires

In order to assess the effects of the experiment on the level of tiredness, boredom, alertness and tiredness of the eyes, a 2 (Phase: pre- and post-training) × 4 (Questions) × 2 between-subject (Groups: experimental and control) repeated ANOVA test was conducted on the scores gathered in the questionnaires. The analysis were followed by *post-hoc* comparisons assessing within-group changes for each group (paired *t*-tests), and between-group differences (one way ANOVAs).

#### 2.5.2. Pre- and Post-training Continuous RDM Task

The response time (RT) was recorded for the correctly responded trials from the onset of the coherent motions until the correct button was pressed by the subject. To consider a response as correct, it must be the first button pressed after the onset and before the end of the coherent motions. Subsequently, the accuracy of each phase was calculated as the number of the correct responses over the total number of targets. For each subject, the mean of RT and the accuracy were calculated in both pre- and post-training phases.

In addition to the above-mentioned behavioral measures, the alpha power (7–12 Hz) of the 500 ms preceding the onset of each correctly responded coherence motion was calculated across centroparietal electrodes (i.e., C3, Cz, C4, P3, Pz, P4). Then the post-training to pre-training ratio of alpha was calculated by dividing the corresponding averaged pre-stimulus alpha powers. The pre-stimulus alpha power is known as a measure of attention engagement and visual awareness (Mathewson et al., 2009; Bengson et al., 2012). Several previous studies including those focusing on the RDM task (O'Connell et al., 2009; Kelly and O 'connell, 2013) reported a link between changes in the pre-stimulus alpha power and variations in RT or accuracy. In this study, the alpha power was calculated after band-pass filtering the signal from 7 to 12 Hz using a zero-phase Butterworth filter. Thereafter, the alpha power of the desired interval was calculated by averaging the square values of the filtered signal in time-domain.

In order to assess the effects of the proposed neurofeedback training on the RDM performance, 2 (Phase: pre- and post-training) × 2 between-subjects (Group: control and experimental) repeated measures ANOVA tests were separately applied on the mean RTs and the accuracies, followed by *post-hoc* comparisons assessing within-group changes for each group (paired *t*-tests), and between-group differences (one way ANOVAs). Moreover, a one way ANOVA was applied on the pre-stimulus alpha ratios to examine between-group differences.

#### 2.5.3. Training Phase: P300-Based Speller Task

##### 2.5.3.1. BCI-speller performance

We compared the performance of the participants in the control and experimental groups in terms of the classification accuracy in the evaluation stage, the first run of the training stage and the after-training stage where the number of flashes per row/column was same across all the participants. During the 2*nd* run to the 4*th* or 5*th* runs of the training stage, the number of flashes per row/column was different from participant to participant depending on participants' performance. So we could not directly compare the classification accuracies of these runs across the two groups. Alternatively, We compared the two groups in terms of the number of flashes per row/column for each participants in the 2*nd*, 3*rd*, and the 4*th* runs of the training stage where different participants received different number of flashes according to their BCI performance. One way ANOVA tests were applied on the classification results and the number of flashes to examine between-group differences.

##### 2.5.3.2. Changes in ERP components of EEG data

To evaluate whether the provided feedback changes EEG patterns in the P300-based speller task, we focused on the total powers of the target and non-target trials calculated from 150 ms to 550 ms after the onset of the stimuli. We considered this particular time interval since it was the one used for classification and subsequently providing feedback. Importantly, an increase in the power of the target trials indicates an enhancement in the ERP components presenting at 150–550 ms after the onset of stimuli due to attending better to the target flashes. Similarly, a decrease in the total power of the non-target trials was interpreted as the subject being less distracted by the non-target flashes.

Following this line of argument, the ratio of changes in the power of the target(non-target) trials when transferring from the calibration and evaluation stages to the training stage was calculated for each subject as follows. The average power of the target(non-target) trials in the training stage was calculated across the centroparietal electrodes, and it was divided by the average power of the target(non-target) trials from the same electrodes in the calibration and evaluation stages. In this manuscript, the obtained value is called *training-to-calibration* target(non-target) ratio. Similarly, the ratio of changes in the power of target(non-target) trials when transferring from the calibration and evaluation stages to the after training stage was calculated for each subject as follows. The average power of the target(non-target) trials in the after training stage was calculated across the centroparietal electrodes. Thereafter, the obtained value was divided by the average power of target(non-target) trials from the same electrodes in the calibration and evaluation stages. In this manuscript, the obtained value is called *after-training-to-calibration* target(non-target) ratio.

A 2 (Trials: target and non-target) × 2 (Stage: training-to-calibration ratios and after-training-to-calibration ratios) × 2 between-subject (Groups: experimental and control) repeated ANOVA test was conducted followed by the following *post-hoc* tests. To examine the changes in the powers of the trials at the training stage due to the feedback, a 2 (Trial: target and non-target) × 2 (Group: experimental and control) repeated ANOVA test was performed on the training-to-calibration ratios. In addition, to evaluate if after the feedback training stage, when no feedback was provided to both groups, the changes in the power of trials were still different between the groups, a 2 (Trial: target and non-target) × 2 (Group: experimental and control) repeated ANOVA test was performed on the after-training-to-calibration ratios. The repeated ANOVA tests were followed by *post-hoc* comparisons assessing within-group changes for each group (paired *t*-tests), and between-group differences (one way ANOVAs).

##### 2.5.3.3. Time/frequency analysis

To better understand which frequency rhythms are more responsible for the changes in the target and non-target trials when transferring from the calibration and evaluation stages to the training stage, the grand mean Event-Related Spectral Perturbation (ERSP) images (Makeig et al., 2004) were plotted at Pz. ERSP is a 2-D (frequency-by latency) image of average changes in the spectral power (in dB) from a baseline. Calculating an ERSP typically requires computing the power spectrum over a sliding latency window, then correcting the baseline by subtracting the pre-stimulus power spectrum, and finally averaging across all the data trials.

Based on the ERSP figures and our prior knowledge on the relationship between alpha rhythm and attention, the changes in the power of the alpha rhythm in non-target trials across centroparietal electrodes were further analyzed using statistical tests. More precisely, the non-target alpha power was calculated for a 150 ms period following non-target stimuli that were not presented immediately after a target stimulus. The *training-to-calibration alpha ratio* was then calculated by dividing the average alpha power of the non-target trials of the training stage to that of the calibration and evaluation stages. Similarly, *after-training-to-calibration alpha ratio* was calculated by dividing the average alpha power of the non-target trials from the after-training stage to the corresponding one from the calibration and evaluation stage. To examine between-group differences, one way ANOVAs were applied on the training-to-calibration as well as the after-training-to-calibration alpha ratios.

#### 2.5.4. Impact of the Training Phase on Continuous RDM Performance

We hypothesized that the performance of the proposed neurofeedback training task can influence the attentional substrate in the continuous RDM task. To test this hypothesis, we investigated whether or not the important features elicited while performing the P300 speller task correlate with the parameters presenting attentional substrates in the continuous RDM task. Thus, the training-to-calibration and after-training-to-calibration ratios of target and non-target trials, and training-to-calibration and after-training-to-calibration alpha ratios of non-target trials were considered as features presenting the performance in the P300 speller task. Subsequently, for the continuous RDM task, the pre- to post training ratios of RTs, the accuracies and the pre-stimulus alpha power were calculated by dividing the results of the post-training phase to the corresponding results of the pre-training phase.

The Pearson's correlation coefficient was used when the assumption of normality of the distributions was met; Otherwise the non-parametric Spearman's correlation test was applied.

### 2.6. Impact of Previous BCI Experience on Performance in RDM and BCI Tasks

The RDM and P300-BCI performance were compared across participants with/without BCI experience to find out if previous P300-BCI experience could influence the results. Thus, a 2 (Phase: pre- and post-training) × 2 between-subjects (Group: with and without BCI experience) repeated measures ANOVA tests were applied on the mean RTs and the accuracies in RDM task, followed by *post-hoc* comparisons. One way ANOVA tests were applied on the BCI classification results of the evaluation stage, the first run of the training stage and the after-training. Moreover, one-way ANOVA test were applied on the number of flashes in the 2*nd*, 3*rd*, and 4*th* runs of the training stagein the to further examine between-group differences.

## 3. Results

### 3.1. Questionnaire

The average scores obtained from the questionnaires are presented in Table 1. A 2 (Phases) × 4 (Questions) repeated Anova with 2 between-subject groups showed that there is a significant main effect of Phase on the questionnaire scores [*F*_(1, 26)_ = 13.97, *p* = 0.001]. However, no significant interactions were observed between the groups and Phases [*F*_(1, 26)_ = 0.1, *p* = 0.75]. This means the changes in the scores were not different across the two groups when transferring for the pre-experiment phase to the post-experiment phase. The *post-hoc* analysis of the pre- and the post-experiment scores further confirmed that both the control and experimental groups were not significantly different in terms of tiredness, alertness, boredom, and tiredness of the eyes. Furthermore, the within-group comparisons revealed that doing the experiment has significantly increased the tiredness of the participants in both control [*t*_(13)_ = −6.28, *p* < 0.001 (two-tailed)] and experimental [*t*_(13)_ = −3.99, *p* = 0.008 (two-tailed)] groups respectively. However, performing the experiment did not lead to a significant change in the level of alertness, boredom and the eyes tiredness across the two groups.

**Table 1:** The average scores to the questions in the questionnaire obtained in the pre- and post-experiment phases.

	**Pre-experiment scores**		**Post-experiment scores**	
**Question**	**Control**	**Experimental**	**Control**	**Experimental**
Tiredness	3.14 (2.18)	3.28 (2.2)	6.00 (1.56)	4.57 (2.47)
Alertness	6.57 (2.21)	7.00 (2.35)	5.85 (1.61)	6.85 (2.17)
Boredom	3 (1.46)	3.71 (1.32)	2.71 (1.63)	2.57 (1.65)
Tiredness of eyes	4.57 (2.14)	3.21 (2.29)	5.42 (2.28)	4.64 (2.59)

*The scores were on a 10-point scale ranging from low to high. The values in the parentheses refer to standard deviation*.

### 3.2. Pre- and Post-training Continuous RDM Task

The mean RTs and accuracies for the continues RDM task in both pre- and post-training phases are presented in Table 2.

**Table 2:** The average response time in milliseconds and accuracy in percentage for each group obtained over the continues RDM task performed at the pre- and post-training phases.

	**Pre-training**		**Post-training**	
**Group**	**RT(std)**	**Acc(std)**	**RT(std)**	**Acc(std)**
Control	906 (150)	85.5 (12.6)	907 (164)	87.4 (11.8)
Experimental	903 (275)	89.1 (12.8)	814 (217)	91.0 (9.6)
Between-groups *P*-value	0.37	0.454	0.03	0.32

*RT, ACC, and std denote response time, accuracy and standard deviation, respectively*.

#### 3.2.1. RT Data

The log transformation was applied on mean RTs to normalize the distributions. One-way ANOVA testing of the log of the mean RTs obtained in the pre-training phase disclosed no significant difference between the two groups prior to the training [*F*_(1, 26)_ = 0.83, *p* = 0.37]. A Phase × Group ANOVA test revealed that both the effect of Phase on RTs [*F*_(1, 26)_ = 3.58, *p* = 0.07] and the interaction of Phase and Group [*F*_(1, 26)_ = 3.20, *p* = 0.08] were tending to significance. Interestingly, the paired *t*-test revealed a significant decrease in the mean RTs in the experimental group [*t*_(13)_ = 2.43, *p* = 0.03 (two-tailed) from the pre-training phase to the post training phase yielded]. In contrast the change in RTs was not significant in the control group [*t*_(13)_ = 0.08, *p* = 0.93 (two-tailed)]. Furthermore, in the post-training phase the RTs were significantly different between the two groups [*F*_(1, 26)_ = 5.12, *p* = 0.03].

#### 3.2.2. Accuracy Data

One-way ANOVA on the accuracies obtained in the pre-training phase indicated that there was no significant difference between the two groups prior to the training [*F*_(1, 26)_ = 0.58, *p* = 0.454]. A Phase × Group ANOVA test revealed neither the effect of Phase on the accuracy [*F*_(1, 26)_ = 1.30, *p* = 0.26] nor the interaction of Phase and Group [*F*_(1, 26)_ < 0.001, *p* = 1] were significant. Consequently, as expected, the increase in the average accuracy when transferred from the pre-training phase to the post-training phase was not significant in either the control [*t*_(13)_ = −0.69, *p* = 0.50 (two-tailed)] or the experimental [*t*_(13)_ = −1.0, *p* = 0.33 (two-tailed)] groups.

#### 3.2.3. Pre-stimulus Alpha Ratios

Finally, transferring from the pre-training to the post-training phase yielded an average reduction of %5 and increase of %11 in the pre-stimulus alpha ratios of the experimental and control groups respectively. The one way ANOVA confirmed that the pre-stimulus alpha ratios are significantly different between the two groups [*p* = 0.05, *F*_(1, 26)_ = 4.00].

### 3.3. Training Phase: P300-Based Speller Task

#### 3.3.1. BCI-Speller Performance

In our study, for 26 participants out of 28, the BCI classification models trained using the calibration data collected during spelling the two words “the” and “quick” achieved satisfactory accuracy in the evaluation stage, since they correctly identified minimum 2 out of 3 letters of the word “dog.” For the remaining two participants (one from each group), the classification model was re-calibrated by adding the EEG data collected from spelling “dog.” The new re-calibrated models were evaluated by spelling the word “fox.” For both two participants, the re-calibrated model achieved the satisfactory accuracy in spelling the new word. Thus, in this study, all the participants were eligible to move to the training stage.

Comparing the evaluation accuracies of the selected calibration models, on average, the experimental and the control groups achieved 97.23 and 92.86% BCI classification accuracies, respectively [*t*_(26)_ = 0.593, *P* = 0.558].

In the first run of the training stage, where both groups spelled the word “beautiful” with 10 flashes per row/column, on average the experimental group achieved 91.16% whereas the control group achieved 82.62% accuracy in correctly spelling the letters. However, there was no statistical difference between the classification results of the two groups [*t*_(26)_ = 1.46, *p* = 0.155]. This finding is aligned with the results of Arvaneh et al. (2015). In Arvaneh et al. (2015), we found that the effect of feedback on P300-speller accuracy was not significant when the number of flashes per row/column was more than 6. The average number of flashes per row/column used in the 2*nd*, 3*rd*, and 4*th* run of the training stage were respectively 6.78, 5.21, and 4.14 for the experimental group and 7.64, 6.21, and 5.00 for the control group. However, again we did not observe any significant differences between the two group.

Finally, in the after-training stage, the experimental group outperformed the control group in terms of BCI classification accuracy by an average of 10% (i.e., 92.25 vs. 82.85%). However, this outperformance was not significant [*t*_(17.56)_ = 1.25, *p* = 0.22].

#### 3.3.2. Changes in ERP Components

**Figure 4:**
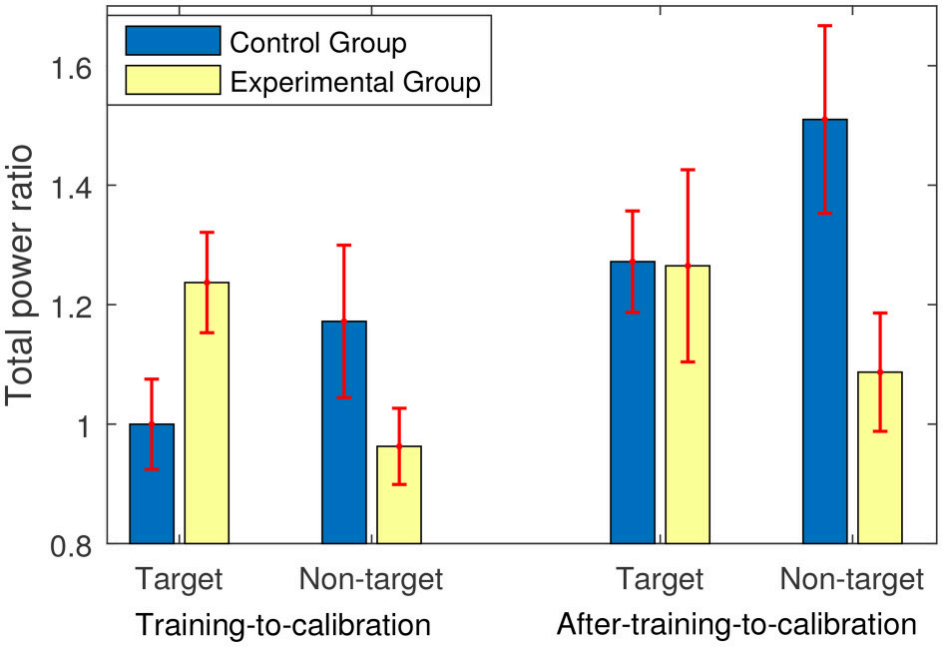
The power ratio of either training or after-training stage to calibration and evaluation stages. The power ratios were calculated for the target and the non-target trials separately.

Figure 4 shows the changes in average powers of the target and non-target trials in the training and after-training stages compared to the calibration and evaluation stages. In fact, a ratio greater than one presents an increase in the average power, and a ratio less than one indicates a decrease in the average power compared to the average power of the corresponding trials in the calibration and evaluation stages. As shown in Figure 4, for the experimental group who received feedback, the training stage yielded on average 23.7% increase in the power of the target trials and 4% decrease in the power of the non-target trials. In contrast, for the control group who did not receive any sort of feedback, the training stage yielded on average less than 0.01% decrease in the power of the target trials and a 17% increase in the power of the non-target trials.

Conducting a 2 (Trials: target and non-target) × 2 (Stage: training-to-calibration ratios and after-training-to-calibration ratios) × 2 between-subject (Groups: experimental and control) repeated ANOVA test revealed a significant main effect of Stage [*p* = 0.005, *F*_(1, 26)_ = 9.63]. Moreover a significant interaction between Trials and Groups [*P* = 0.003, *F*_(1, 26)_ = 11.07] was revealed and a tending to significant interaction between Stages and Groups [*P* = 0.073, *F*_(1, 26)_ = 3.49] were observed. These findings suggest that transferring from the training stage to the after-training stage yielded different powers of trials across the groups. Moreover, changes in the power of the target trials and non-target trials were quantitatively different across groups.

*Post-hoc* analyses on the training-to-calibration ratios revealed a significant interaction between Groups and Trials [*F*_(1, 26)_ = 9.63, *p* = 0.005]. This suggests a quantitative difference between the changes in the powers of the target and the non-target trials across experimental and control groups. Moreover, the training-to-calibration target ratios were significantly different between the two groups [*F*_(1, 26)_ = 4.75, *p* = 0.03], whereas no significant difference was observed between the training-to-calibration non-target ratios [*F*_(1, 26)_ = 2.30, *p* = 0.14].

**Figure 5:**
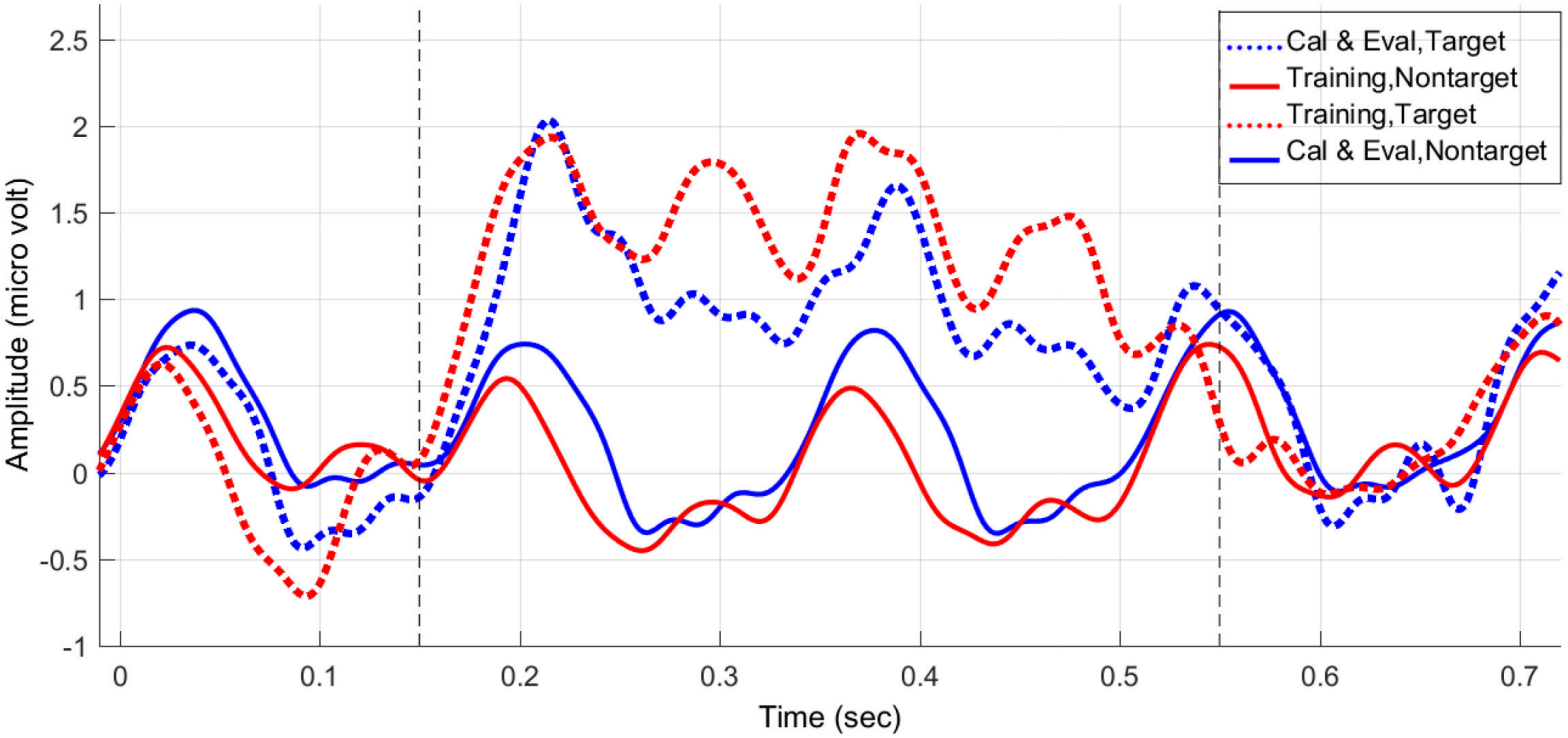
The average stimulus-locked ERPs of one subject in the experimental (feedback) group generated during the P300-based speller task, plotted at Pz.

As an example, Figure 5 shows the average stimulus-locked EEG responses of one subject from the experimental group over the calibration and evaluation stages vs. the training stage. Comparing the average stimulus-locked EEG responses, we can clearly observe an average increase in the power of the target trials and an average decrease in the power of non-target trials of the training stage compared to the calibration and evaluation stages (please see 150–550 ms after the onset of the stimuli). Thus, as Figure 5 elaborates as part of our statistical results, unlike the control group the experimental group was able to successfully enhance the ERP components during target trials while attenuating the evoked response power generated to non-target trials during the feedback session.

Back to Figure 4, we can also see that at the after-training stage where no feedback was provided, both groups showed similar increases in the average power of the target trials compared to the calibration and evaluation stages (i.e., around 27%). Since the participants knew that the after-training stage is the last stage to perform, they were most-likely motivated to do their best. Thus, although no feedback was provided both groups generated enhanced ERP components for the target trials compared to the calibration and evaluation stages. Furthermore, the learning effects can be another reason for the observed improvement in the power of the target trials in both groups. Exploring further, an average increase of 51% was observed in the power of the non-target trials in the control group, whereas this increase was only 8% in the experimental group. *Post-hoc* analyses focusing on the after-training-to-calibration ratios showed a significant interaction between Trials [*F*_(1, 26)_ = 5.24, *p* = 0.03] and Groups. Interestingly, a one way ANOVA test showed that the after-training-to-calibration non-target ratios are significantly different between the two groups [*F*_(1, 26)_ = 5.68, *p* = 0.02], whereas no significant difference was observed between the after-training-to-calibration target ratios [*F*_(1, 26)_ = 0.001, *p* = 0.97]. This shows that immediately after training when no feedback was provided the experimental group was still better than control group in terms of generating more neutral EEG signals in response to the non-target trials.

#### 3.3.3. Time/Frequency Analysis

**Figure 6:**
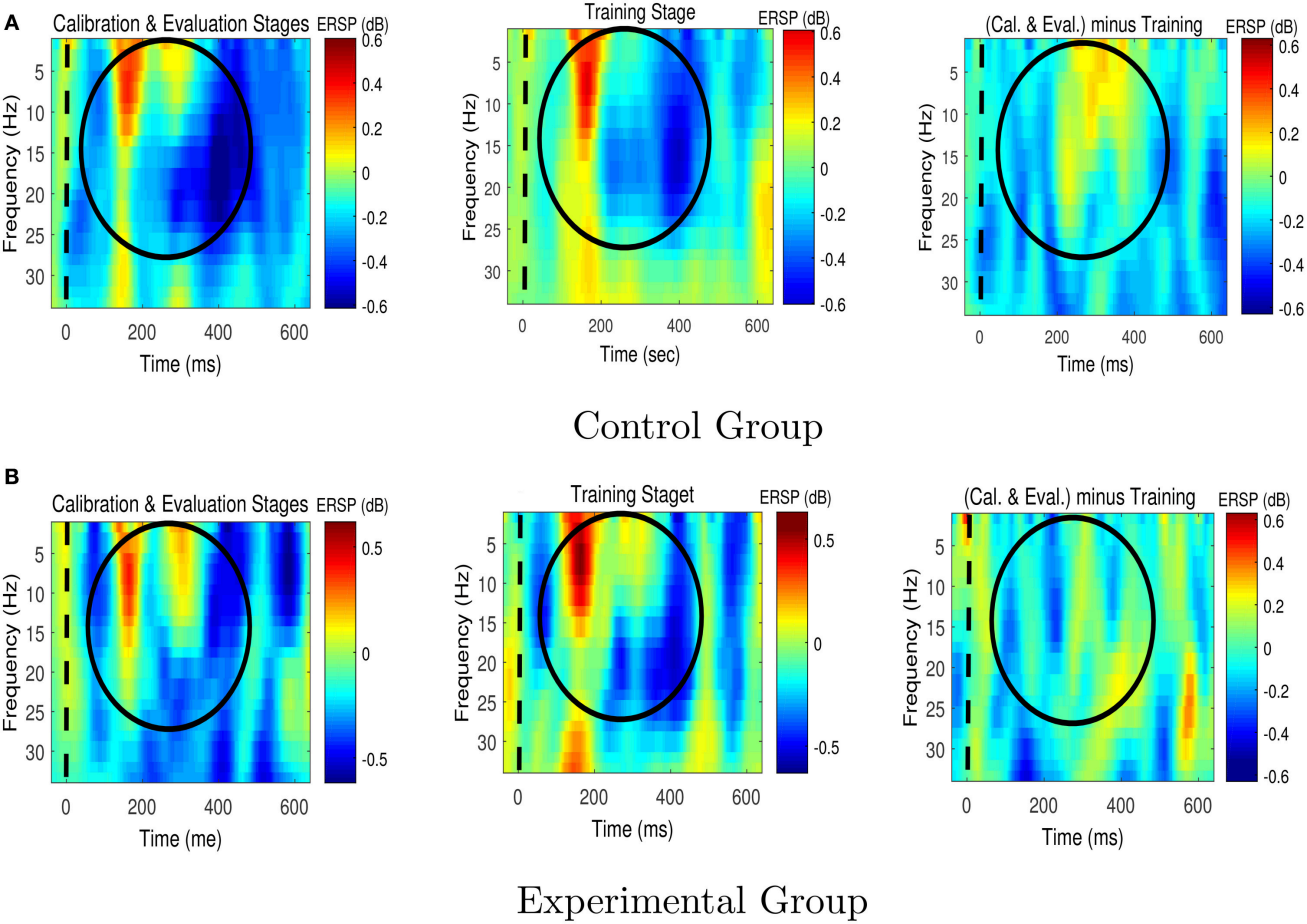
The Event-Related Spectral Perturbation (ERSP) images for the target trials for **(A)** the control group, and **(B)** the experimental group. The ERSP images were plotted at Pz. The dashed lines denote the cue time. Cal. and Eval. denote calibration and evaluation, respectively.

**Figure 7:**
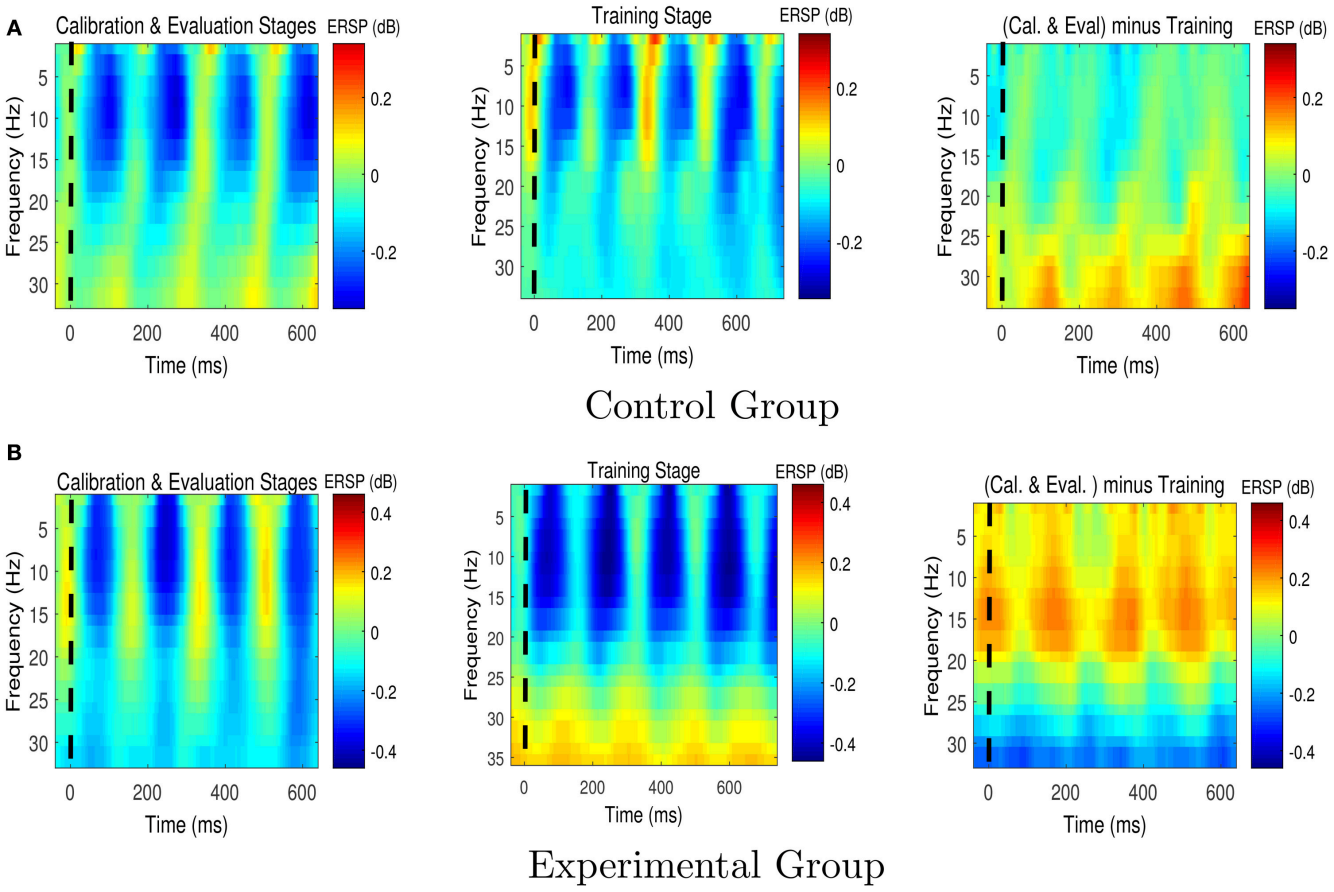
The Event-Related Spectral Perturbation (ERSP) images for the non-target trials for **(A)** the control group, and **(B)** the experimental group. The ERSP images were plotted at Pz. The dashed lines denote the cue time. Cal. and Eval. denote calibration and evaluation, respectively.

Figures 6, 7 respectively present the ERSP images obtained by grand averaging the target and non-target trials, time locked to the cue time, recorded at Pz. In the ERSP images plotted using the calibration and evaluation stages as well as the training stage, red indicates enhancement of the power with respect to the pre-cue baseline, and blue indicates suppression of the power with respect to the pre-cue baseline. In the calibration-and-evaluation minus training ERSP images, red indicates higher power in the calibration and evaluation stages compared to the training stage, and blue indicates higher power in the training stage compared to the calibration and evaluation stages.

As shown in Figure 6, in the experimental group transferring from the calibration and evaluation stages to the training stage yielded an increase in theta and alpha power around 150–400 ms after the presentation of target stimuli, whereas in the control group the brain signals presented the opposite characteristics, i.e., a reduction in theta and alpha powers.

**Figure 8:**
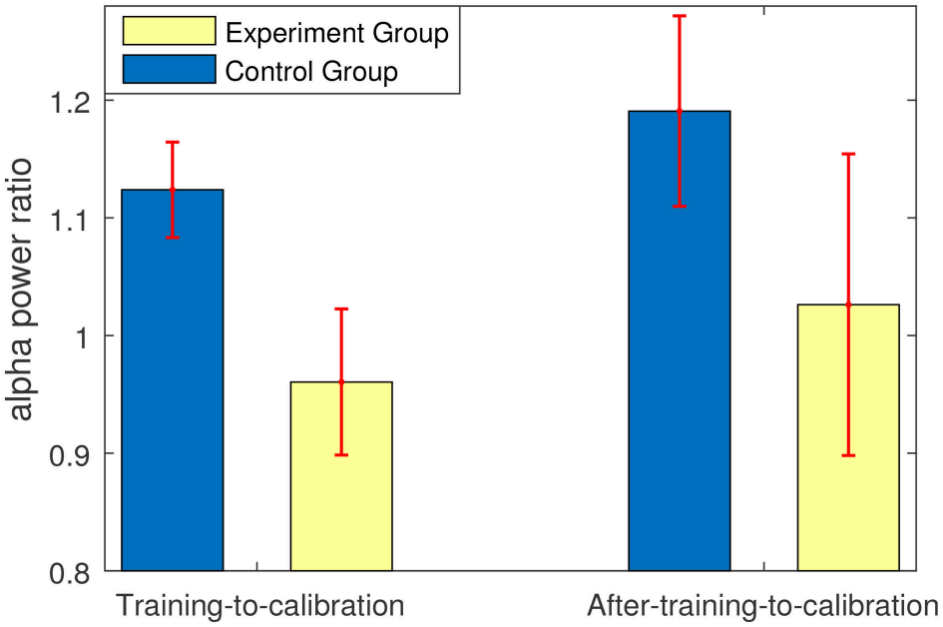
Ratios of alpha power in either training or after-training stages to that of the calibration and evaluation stages. The alpha powers were calculated only for non-target trials.

Figure 7 shows that compared to the control group, the experimental group who received feedback during training stage were able to suppress the non-target alpha power more. Similarly, Figure 8 presents average changes in non-target alpha power obtained from centro-parietal electrodes when transferring from the calibration and evaluation stages to either the training or the after-training stage. As shown in Figure 8, transferring to the training stage yielded an average %4 decrease in the alpha power of the experimental group, whereas an average %12 increase was observed for the control group. The one way ANOVA test on the training-to-calibration alpha ratios revealed a significant difference between the two groups [*p* = 0.037, *F*_(1, 26)_ = 4.79]. Subsequently, transferring to the after-training stage yielded an average %2 and %19 increase in the alpha power of the non-target trails for the experimental and control groups respectively. Although the control group presented an increase in alpha power, the after-training-to-calibration ratios of alpha were not significantly different between the two groups [*p* = 0.288, *t*_(26)_ = −1.08].

### 3.4. Impact of the Training Phase on Continuous RDM Performance

No significant correlation was observed between the features presenting behavioral changes in the RDM task (i.e., pre-to-post training ratios of RTs, and pre-to-post ratios of the accuracies) and the P300-based speller neural features named in section 2.5.4. However, a significant positive correlation was observed between the pre-to-post training pre-stimulus alpha ratios in the continuous RDM task and the training-to-calibration alpha ratios of non-target trials in the P300-speller task (*r* = 0.319, *p* = 0.049). Interestingly, we also observed a significant correlation between the pre-stimulus alpha ratios and the RT ratios in the continuous RDM task (*r* = 0.32, *p* = 0.048).

### 3.5. Impact of Previous BCI Experience on RDM and BCI Performance

In the pre-training phase of the RDM task, on average those who had previous P300-BCI experience achieved mean RT of 855±206 ms and accuracy of 89.33%, whereas those who were naive to BCI achieved mean RT of 887±234 ms and accuracy of 82.95%. Interestingly, when transferring to the post-training phase, a greater improvement in the mean RT was observed for those who had previous P300-BCI experiment. The participants with previous BCI experience achieved 757±223 ms average mean RT and 92.5% accuracy in the post-training phase, whereas those without BCI experience obtained on average 875.9±209 mean RT and 86.11% accuracy. The ANOVA tests revealed neither significant main effects nor significant interactions on the results. Moreover, there were no significant differences between the two groups in terms of mean RTs and RDM accuracies across the two phases.

All 6 participants with BCI experience were able to achieve a satisfactory BCI calibration model only using EEG data recorded during spelling the words “the” and “quick.” on average, those with and without BCI experience respectively achieved average BCI classification accuracies of 100 and 95.46% in the evaluation stage, 81.22 and 88.43% in the first run of the training stage, and 88.57 and 87.27% in the after-training stage. On average, in the 2*nd*, 3*rd*, and 4*th* runs of the training stage 6.33, 5, and 4.16 number of flashes were presented to the participants with BCI experience, whereas the participants without BCI experience received 7.45, 5.9, and 4.68 flashes respectively. However, in none of these stages and runs, the classification accuracies and/or number received flashes were significantly different between the two groups.

Finally we would like to emphasize that although the above-mentioned results are interesting, we are not be able to make any conclusions as there were only 6 participants with BCI experience against 22 participants without BCI experience. More participants with BCI experience evenly distributed in both the experimental and control group are needed in order to make a reliable and unbiased analysis.

## 4. Discussion and Conclusion

This paper proposed a modified version of the P300-based speller BCI as a neurofeedback training tool. The effectiveness of the proposed P300-based neurofeedback tool on short time scales (i.e., only around 30 min training) was investigated from two points of view, namely (1) changes in EEG components during the training session, and (2) immediate pre-/post-training changes in cognitive performance and EEG.

Performing the proposed P300-based neurofeedback training in the experimental group where feedback was provided led to enhancement of ERP components during the target trials, as well as the generation of more neutral EEG signals in response to non-target trials. Our results suggest that providing feedback while reducing the number of flashes per row/column encourages the users to improve/maintain their performance by generating more discriminable EEG ERPs in response to the target stimuli vs. the non-target stimuli. This was achieved by enhancement in the ERP components of the target trials (i.e., 150–550 ms after the onset of stimuli which includes P300) as well as attenuation in the corresponding ERP components of the non-target trials. These findings were further supported by our results obtained from analysing the alpha power of the non-target trials. As the alpha suppression reflects attentional processes where relative desynchronization in the alpha band in areas processing potential target information reflects preparatory enhancement (Foxe and Snyder, 2011). Changes in the alpha power of the non-target trials suggests that the experimental group were distracted less by the irrelevant stimuli during the training phase.

The results of performing P300-based BCI immediately after training was also interesting. Our results showed that compared to the control group the experimental group were less distracted by the non-target trials although no feedback was provided in this stage.

Finally our results for the RDM task performed immediately before and after P300-based BCI use, showed that the experimental group had a significant improvement in RT in the post-training phase compared to the pre-training phase, whereas the control group did not show such improvements. Similarly, transferring from pre- to post-training phase yielded a significant reduction in pre-stimulus alpha power of the experimental group compared to the control group. Indeed, changes in the pre-stimulus alpha was significantly correlated with the changes in RT across all the participants. Interestingly, our finding revealed that the pre-to-post training changes in EEG were linked to the P300-BCI task through a significant correlation observed between pre-to-post training pre-stimulus alpha ratio in RDM and training-to-calibration alpha ratio of non-target trials of the P300-BCI task. However, we did not find a direct link between changes in RTs in pre-post training phases and changes in EEG components during the P300-BCI task.

In terms of the experimental time, the proposed P300-based neurofeedback system has a similar calibration time to a conventional P300-speller where 12 flashes per row/column are used for spelling each letter. As explained in section 2.3.4, in the neurofeedback training stage, the first run used 10 flashes per row/column while in the subsequent runs the number of flashes were changed based on the user's performance in order to keep the accuracy above 66%. For all our participants in the experimental group, this strategy led to reducing the number of flashes per row/column, whereby a few participants spelled the last word using only 1–2 flashes per row/column. Thus, it can be concluded that for spelling same words the experimental time for the proposed P300-based neurofeedback system can be considerably less than the conventional P300-speller where typically 12 flashes per row/column is used for spelling each letter.

In conclusion, this paper demonstrates a neurofeedback training method which works based on EEG components in the time domain (i.e., P300). The proposed neurofeedback training framework is entertaining and fun due to its game-like structure. Importantly, unlike other available neurofeedback training methods, the proposed method does not require users to undergo several “trial and error” sessions in order to develop a strategy for controlling the signal feature. Since the P300 can be generated easily and naturally in response to target stimuli, after a relatively short period (10–12 min) collecting the data, the user is able to interact usefully with the proposed training tool. The level of difficulty of the proposed method is adapted based on the user's performance to avoid frustration or boredom.

This paper in fact presented a feasibility and proof of concept study. Much remains to be investigated, including long term effects of this training. Further insight can be gained by applying the proposed P300-based neurofeedback training tool over multiple sessions across larger groups of participants. The proposed neurofeedback training tool was tested using young healthy participants who are known having relatively high performance in P300-based BCI and RDM tasks. That would be interesting to present the efficacy of the proposed P300-based neurofeedback training for those who are at risk of attention problems and those who have lower attention capacities, such as elderly. Moreover, the usability and acceptance of the proposed training tool needs to be investigated if targeted by other populations.

## Author Contributions

MA designed the experiment, developed the real-time neurofeedback interface, collected data, contributed to analysis and interpretation of data, and wrote the initial draft of the manuscript. TW have contributed to data analysis and interpretation, and critically reviewed the manuscript. IR contributed in designing the experiment and analysing and interpretation of data. The final version of the manuscript was approved by all authors. TW and IR are joint senior authors.

### Conflict of interest statement

The authors declare that the research was conducted in the absence of any commercial or financial relationships that could be construed as a potential conflict of interest.
